# Novel Orthobunyavirus Identified in the Cerebrospinal Fluid of a Ugandan Child With Severe Encephalopathy

**DOI:** 10.1093/cid/ciy486

**Published:** 2018-06-09

**Authors:** Arthur W D Edridge, Martin Deijs, Ruth Namazzi, Cosimo Cristella, Maarten F Jebbink, Irma Maurer, Neeltje A Kootstra, Linda R Buluma, Job B M van Woensel, Menno D de Jong, Richard Idro, Michael Boele van Hensbroek, Lia van der Hoek

**Affiliations:** 1 Laboratory of Experimental Virology, Department of Medical Microbiology, Amsterdam, The Netherlands; 2 Global Child Health Group, Emma Children’s Hospital, Academic Medical Center, Amsterdam, The Netherlands; 3 Department of Paediatrics and Child Health, Mulago Hospital, Makerere University College of Health Sciences, Kampala, Uganda; 4 Laboratory of Viral Immune Pathogenesis, Department of Experimental Immunology, Academic Medical Center, Amsterdam, The Netherlands

**Keywords:** virus discovery, orthobunyavirus, CNS infection, Uganda, encephalitis

## Abstract

A Ugandan child with an unexplained encephalitis was investigated using viral metagenomics. Several sequences from all segments of a novel orthobunyavirus were found. The S-segment, used for typing, showed 41% amino acid diversity to its closest relative. The virus was named Ntwetwe virus, after the hometown of the patient.

In sub-Saharan Africa, children often present to hospitals with fever and acute alterations of consciousness, yet causes remain unidentified in up to 75% of such cases [1]. Recent insights have indicated that central nervous system (CNS) infections of viral origin are more prevalent than previously thought, and novel viruses may explain some of these unexplained CNS infections [2]. Uganda is considered a hotspot for zoonotic and vector-borne pathogens, where such novel viruses may emerge [3]. In 2016, a Ugandan girl presented to the hospital with a suspected CNS infection for which no pathogen could be identified using conventional diagnostics. Viral metagenomic sequencing was performed to find the causative agent and a novel orthobunyavirus was identified. Orthobunyaviruses are negative-sense RNA viruses consisting of 3 segments: large (L), medium (M), and small (S). They are commonly found in Uganda, are often zoonotic and vector-borne, and are known to cause human CNS infections [4, 5].

## METHODS

Cerebrospinal fluid (CSF) and ethylenediaminetetraacetic acid plasma samples were collected from the patient 2 weeks after onset of symptoms at the Mulago National Referral Hospital in Kampala, Uganda, and stored at −80°C. Viral metagenomic sequencing was performed on the CSF and plasma samples. Viral sequences were extended by genome walking, and the resulting contigs were used to infer an amino acid phylogeny and identity matrix. A reverse transcription quantitative polymerase chain reaction was developed, and the virus was quantified in CSF and plasma. Inflammatory biomarkers were measured in CSF and plasma using a Luminex panel. Details of the assays are provided in the Supplementary Methods.

## RESULTS

### Description of the Patient

**Figure 1.**
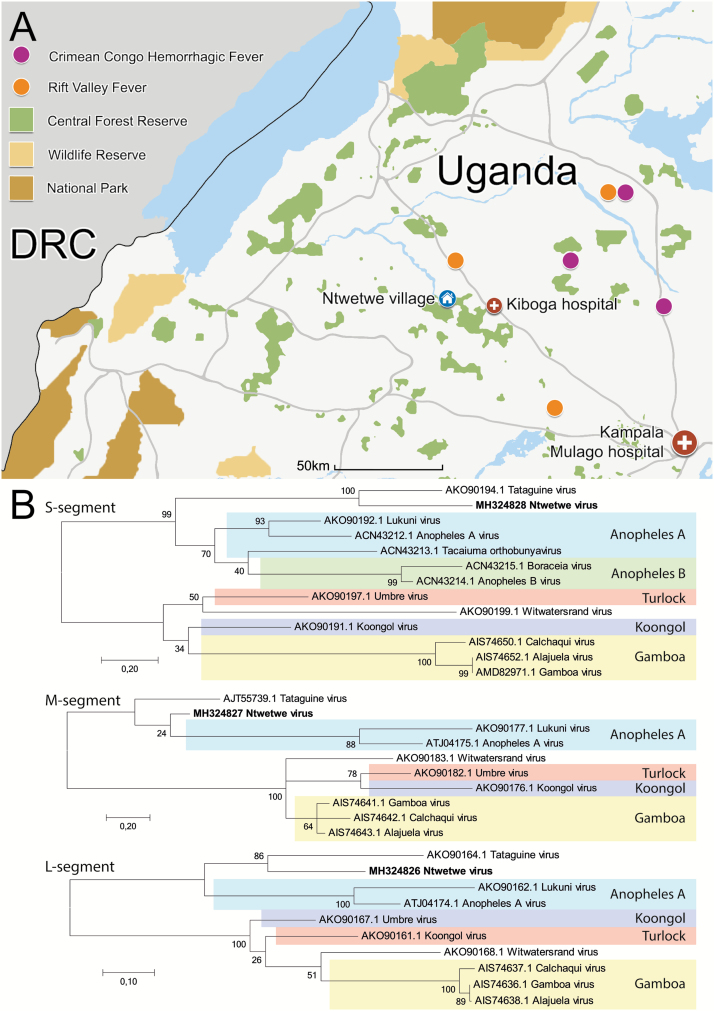
*A*, Map of central and western Uganda. The hometown of the patient, Ntwetwe village, is marked in blue; the hospitals visited by the patient are marked in red. Recent reports of bunyavirus outbreaks reported to ProMED-Mail since November 2017 are marked in purple and orange. Central forest reserves are in green, wildlife reserves are in yellow, and national parks are in brown. *B*, Phylogenetic analysis on amino acid sequences of the N protein (coded by the S-segment), glycoprotein precursor polyprotein (coded by the M-segment), and L protein (coded by the L-segment). The Ntwetwe virus amino acid sequence was based on translated nucleotide sequences of 283, 178, and 936 nucleotides of the S, M, and L segments, respectively. Known serogroups are coded by color. GenBank accession numbers (MH324826, MH324827, MH324828) are provided for each viral segment. Abbreviation: DRC, Democratic Republic of Congo.

In February 2016, a previously-healthy 3-year-old Ugandan girl fell ill in her hometown, Ntwetwe village, and developed a fever, a headache, and abdominal pain (Figure 1). She was given paracetamol and an antimalarial (artemether-lumefantrine) and was seen at 2 local health clinics and at Kiboga district hospital before being referred to Mulago National Referral Hospital in Kampala because of her deteriorating health.

At the time of arrival at Mulago hospital, the patient had been ill for 2 weeks. She presented in a coma (Modified Glasgow Coma Scale 6/15) with prolonged, generalized convulsions that did not respond to 2 doses of first-line anticonvulsants. She had a temperature of 39.2°C, a blood pressure reading of 137/93 mmHg, a heart rate of 198 beats/minute and a respiratory rate of 40 breaths/minute. She displayed decorticate posturing, had absent corneal reflexes, and had papilledema on funduscopy.

On laboratory examination, her haemoglobin level was 13.3 g/dL. CSF analysis showed less than 5 total cells/mm^3^, and normal glucose and protein levels. A malaria blood slide was negative, and the bacterial CSF culture remained negative after 7 days.

The patient was admitted to the High Dependency Unit and treated with amlodipine, intravenous (IV) ceftriaxone, IV metronidazole, and IV phenobarbitone. Nonetheless, she did not regain complete consciousness and left the hospital. She died at home 2 weeks after leaving the hospital.

### Molecular Diagnostics

The severity of outcome elicited a further investigation into the cause of disease. The clinical syndrome of decreased consciousness, fever, and seizures in absence of malaria parasitaemia (ruling out cerebral malaria) were suggestive of an encephalitis [6]. Consequently, real-time polymerase chain reactions (PCRs) of herpes simplex virus 1 and 2, varicella zoster virus, parechovirus, enterovirus, mumps, and rubella virus were performed in CSF, but all were negative. Considering the absent pleiocytosis and normal CSF glucose and protein, a bacterial cause was unlikely, yet to further exclude this cause, real-time PCRs for *S. pneumoniae*, *N. meningitidis*, and *H. influenzae* were performed. These tests also were negative.

### Viral Metagenomics and Genome Walking

To test for an unexpected or unknown virus, a viral metagenomics assay was performed on CSF and plasma, which initially generated 6,947 sequence reads. No known viruses were identified, yet 2 sequence reads (partially overlapping) showed some, although low, amino acid identities (the percentage of matching amino acids) to several orthobunyavirus L-segments. Using genome walking, the L-segment sequence was extended to 936 nucleotides. A PCR developed to target this sequence confirmed the presence of this sequence in both CSF and plasma of the patient.

To acquire additional sequence information and also confirm that no other viral infection occurred in this patient, 2 Illumina MiSeq libraries were subsequently prepared on separate CSF and plasma samples. Among the total of 4.5 million reads, no known virus was found, yet several sequences of the M and S segments of the novel virus were found. Consequently, sequence information from all 3 orthobunyavirus segments was obtained. All showed large genetic variations to their closest relative, the Tataguine virus (Supplementary Table 1). Hence, the virus was deemed novel and named Ntwetwe virus, after the place where the patient fell ill.

### Phylogenetic Clustering and Amino Acid Identities

To determine the phylogenetic clustering of Ntwetwe virus with known orthobunyaviruses, reference sequences from related serogroups were used (Figure 1A). For all segments, Ntwetwe virus clustered within the Anopheles-Tataguine clade (containing the Anopheles A and B serogroups and unclassified Tataguine virus) and related most closely to Tataguine virus. Phylogenetic analysis was also done on the nucleotide level, and yielded similar clustering. The amino acid identity to the closest relative for the S-segment was 59% (see Supplementary Table 2 for a complete S-segment amino acid identity matrix).

### Viral Load Measurements

To determine the virus concentration, a reverse transcription quantitative polymerase chain reaction was performed, which provided a viral load of 3 × 10^2^ RNA copies/mL in CSF and 5 × 10^3^ RNA copies/mL in plasma.

### Immunological Profiling of the Ntwetwe Virus Patient and Reference Cases

To provide additional support for a causative relationship between Ntwetwe virus infection and encephalopathy, a clinically-relevant Ntwetwe virus infection was hypothesized to elicit a host immune response similar to CNS infections with known viral pathogens. A panel of 11 inflammatory biomarkers was measured in CSF and plasma. The concentrations in plasma were compared to those of reference patients with encephalopathies of varying etiologies (see Supplementary Methods).

For plasma, the biomarker profile of the Ntwetwe virus patient was most similar to those of patients with a CNS infection (Supplementary Figure 1 and Supplementary Results).

For CSF, an insufficient availability of sample material of reference cases restricted similar comparisons of biomarker profiles, as described above for plasma. Therefore, the individual inflammatory biomarker concentrations were compared to reference ranges (Supplementary Table 3) [7]. Elevated inflammatory biomarker concentrations were found for interleukin (IL) 4, IL-6, IL-8, monocyte chemoattractant protein 1, monokine induced by γ-interferon, and tumor necrosis factor α. These concentrations are in line with what is generally seen in viral encephalitis, and make another cause (eg, an auto-immune cause) highly unlikely [7]. More details regarding the inflammatory biomarker results are available in the Supplementary Results.

## DISCUSSION

Ntwetwe virus is a novel orthobunyavirus identified in the CSF of a Ugandan child with a suspected CNS infection for which, despite extensive conventional investigations, no known pathogen could be identified. Partial sequence information from all (S, M, and L) Ntwetwe virus segments was obtained, and showed considerable genetic distance to currently known viruses. According to the International Committee on Taxonomy of Viruses, the amino acid sequences of the nucleocapsid proteins differ by more than 10% for distinct orhobunyavirus species [8]. The Ntwetwe virus nucleocapsid protein, coded for by the S-segment, showed a 41% protein distance to its closes relative, Tataguine virus.

The clinical presentation and host response of the blood and CNS comparments of the described patient were highly suggestive of a viral encephalitis. The decorticate posturing and autonomic dysregulation were likely suggestive of a severe brain impairment that may have been caused by brainstem herniation or cerebral haemorrhaging, which both are known to occur during severe forms of encephalitis [9]. Alternatively, direct neuronal damage by the infection or immunopathology may have contributed to the severe encephalopathy. Because Ntwetwe virus was the only virus identified in this patient, a viral encephalitis, elicited by the Ntwetwe virus was the most likely cause of disease.

Thus far, the patient described here is the only patient identified with a Ntwetwe virus infection. The prevalence of Ntwetwe virus infection, therefore, currently remains unknown. Phylogenetically, Ntwetwe virus clusters with the Anopheles-Tataguine clade, of which all viruses are transmitted by *Anopheles* mosquitoes [10], making Ntwetwe virus a likely arbovirus. *Anopheles* mosquitoes are highly prevalent in Uganda [11], and may thus increase the likelihood of human infections. Additionally, many orthobunyaviruses have non-human mammalian reservoirs [12]. Western Uganda is exceptionally biodiverse, with a high concentration of national parks, wildlife reserves, and forest reserves where such animal reservoirs may exist (Figure 1A) [13]. Besides, rapid growth in the human population and deforestation have recently increased human interactions with such animals in exactly this region [14], increasing the chances for zoonoses to emerge, as demonstrated by recent outbreaks of Crimean Congo Hemorrhagic fever virus and Rift Valley fever virus, 2 zoonotic bunyaviruses, near Ntetwe village (Figure 1A) [15]. Consequently, because of the potential human exposure and severe disease of the patient, we believe there is an urgent need to investigate the prevalence of Ntwetwe virus infections.

## Supplementary Data

Supplementary materials are available at *Clinical Infectious Diseases* online. Consisting of data provided by the authors to benefit the reader, the posted materials are not copyedited and are the sole responsibility of the authors, so questions or comments should be addressed to the corresponding author.

## Supplementary Material

Supplementary Table 1

Supplementary Table 2

Supplementary Table 3

Supplementary Table 4

Supplementary Table 5

Supplementary Table 6

Supplementary Table 7

Supplementary Methods

Supplementary Results

Supplementary Figure 1

## Notes

***Acknowledgments.*** The authors thank the pediatric accident and emergency ward staff of the Mulago hospital in Uganda for gathering the clinical information from the patient. We also thank the Mulago hospital staff, the pediatric research ward staff at Queen Elizabeth Hospital in Malawi, and the pediatric staff of Kigali Central University Hospital in Rwanda for providing the samples used for the inflammatory biomarker analysis. We thank C. M. Kinsella for developing the bioinformatic pipeline.

***Financial support.*** This work was supported by the Academic Medical Center (PhD scholarship to A. W. D. E.); the Platform for European Preparedness Against (Re-)emerging Epidemics project; the European Union Seventh Framework Programme (grant 602525; statistical methodology by C. C.); Host Switching Pathogens, Infectious Outbreaks and Zoonosis; the European Union Horizon 2020 Innovative Training Networks (grant 721367); and The Netherlands-African Partnership for Capacity Development and Clinical Interventions against Poverty-Related Diseases-Africa Research Initiative and Support program, sponsored by the Dutch Government (clinical study).

***Potential conflicts of interest.*** All authors: No reported conflicts. All authors have submitted the ICMJE Form for Disclosure of Potential Conflicts of Interest. Conflicts that the editors consider relevant to the content of the manuscript have been disclosed.
