# Supplementary material for: Novel Orthobunyavirus Identified in the Cerebrospinal Fluid of a Ugandan Child With Severe Encephalopathy
Source: Clin Infect Dis. 2018 Jun 9;68(1):139–42. doi: 10.1093/cid/ciy486 (PMC6293039; doi:10.1093/cid/ciy486)
Supplement: Supplementary Table 1 [file ciy486_suppl_supplementary_table_1.doc]

| **Supplementary Table 1.** Amino acid identities for Ntwetwe virus compared to viruses in the Anopheles-Tataguine clade. | | | |
| --- | --- | --- | --- |
| **Virus** | **S-Segment (%)** | **M-segment (%)** | **L-segment (%)** |
| AKO90194.1 Tataguine | 59 | 62 | 71 |
| AKO90192.1 Lukuni | 41 | 43 | 60 |
| ACN43212.1 Anopheles A | 39 | 46 | 64 |
| ACN43214.1 Anopheles B | 40 | NA^a^ | NA |
| ACN43215.1 Boraceia | 34 | NA | NA |
| ACN43213.1 Tacaiuma | 41 | NA | NA |
| a: NA = not available because the segments have not been sequenced. | | | |
