# Supplementary material for: Novel Orthobunyavirus Identified in the Cerebrospinal Fluid of a Ugandan Child With Severe Encephalopathy
Source: Clin Infect Dis. 2018 Jun 9;68(1):139–42. doi: 10.1093/cid/ciy486 (PMC6293039; doi:10.1093/cid/ciy486)
Supplement: Supplementary Table 2 [file ciy486_suppl_supplementary_table_2.docx]

| **Supplementary Table 2.** Amino acid identity matrix for the S-segment of Ntwetwe virus compared to related orthobunyaviruses. | | | | | | | | | | | | | | | | | |  |
| --- | --- | --- | --- | --- | --- | --- | --- | --- | --- | --- | --- | --- | --- | --- | --- | --- | --- | --- |
| **Serogroup** | **Virus** | **Ntwetwe (%)** | Tataguine (%) | Anopheles A (%) | Lukuni (%) | Tacaiuma (%) | Anopheles B (%) | Boraceia (%) | Alajuela (%) | Gamboa (%) | Calchaqui (%) | Abbey lake (%) | Bunyamwera (%) | Wyeomyia (%) | La Crosse (%) | Inkoo (%) | Trivittatus (%) |  |
| Ung^a^ | **Ntwetwe virus** | ID^e^ |  |  |  |  |  |  |  |  |  |  |  |  |  |  |  |  |
| Ung | AKO90194.1 Tataguine | 59 | ID |  |  |  |  |  |  |  |  |  |  |  |  |  |  |  |
| Anoph^b^ A | ACN43212.1 Anopheles A | 39 | 41 | ID |  |  |  |  |  |  |  |  |  |  |  |  |  |  |
|  | AKO90192.1 Lukuni | 41 | 41 | 65 | ID |  |  |  |  |  |  |  |  |  |  |  |  |  |
|  | ACN43213.1 Tacaiuma | 41 | 43 | 49 | 52 | ID |  |  |  |  |  |  |  |  |  |  |  |  |
| Anoph B | ACN43214.1 Anopheles B | 40 | 41 | 44 | 50 | 48 | ID |  |  |  |  |  |  |  |  |  |  |  |
|  | ACN43215.1 Boraceia | 34 | 41 | 40 | 46 | 47 | 77 | ID |  |  |  |  |  |  |  |  |  |  |
| Gamboa | AIS74652.1 Alajuela | 30 | 33 | 32 | 31 | 33 | 27 | 26 | ID |  |  |  |  |  |  |  |  |  |
|  | AMD82971.1 Gamboa | 30 | 33 | 32 | 31 | 33 | 27 | 26 | <100 | ID |  |  |  |  |  |  |  |  |
|  | AIS74650.1 Calchaqui | 31 | 32 | 32 | 30 | 32 | 27 | 28 | 80 | 80 | ID |  |  |  |  |  |  |  |
| Bunya^c^ | AIA08882.1 Abbey lake | 30 | 29 | 32 | 32 | 27 | 25 | 24 | 33 | 33 | 32 | ID |  |  |  |  |  |  |
|  | AKX73311.1 Bunyamwera | 30 | 33 | 31 | 34 | 31 | 26 | 26 | 34 | 34 | 34 | 72 | ID |  |  |  |  |  |
|  | AEZ35279.1 Wyeomyia | 35 | 36 | 38 | 39 | 35 | 33 | 28 | 37 | 37 | 35 | 56 | 62 | ID |  |  |  |  |
| Cal enc^d^ | AKP04433.1 La Crosse | 35 | 37 | 39 | 36 | 41 | 32 | 29 | 34 | 34 | 38 | 37 | 35 | 43 | ID |  |  |  |
|  | ANW09503.1 Inkoo | 37 | 37 | 37 | 37 | 35 | 32 | 27 | 37 | 37 | 38 | 35 | 35 | 43 | 82 | ID |  |  |
|  | APA34121.1 Trivittatus | 33 | 38 | 36 | 36 | 33 | 32 | 30 | 34 | 34 | 35 | 38 | 37 | 44 | 74 | 78 | ID |  |
| a: Ung = ungrouped, b: Anoph = Anopheles, c: Bunya = Bunyamwera, d: Cal enc = California encephalitis, e: ID = identical | | | | | | | | | | | | | | | | | | |
