# Supplementary material for: Novel Orthobunyavirus Identified in the Cerebrospinal Fluid of a Ugandan Child With Severe Encephalopathy
Source: Clin Infect Dis. 2018 Jun 9;68(1):139–42. doi: 10.1093/cid/ciy486 (PMC6293039; doi:10.1093/cid/ciy486)
Supplement: Supplementary Table 3 [file ciy486_suppl_supplementary_table_3.doc]

| **Supplementary Table 3** – Concentration of inflammatory biomarkers in CSF. | | | | | | | | | | | |  |
| --- | --- | --- | --- | --- | --- | --- | --- | --- | --- | --- | --- | --- |
| **Sample** | **GM-CSF** | **IL-10** | **IL-4** | **IL-6** | **IL-8** | **IP-10 (CXCL10)** | **MCP-1 (CCL2)** | **MIG (CXCL9)** | **MIP-1α (CCL3)** | **MPO** | **TNF-α** |  |
| CSF | 9.04^a^ | 10.66 | 28.43*^b^ | 100.81* | 928.95* | 78.05 | 2840.74* | 201.69* | 3.87 | 242.01 | 26.12* |  |
| Reference range | (2-340) | (0-29) | (0-3) | (1.35-15) | (5.4-32.3) | (45-1299) | (136-820) | (3-3) | (0.48-6.4) | (NA) | (0-7.5) |  |
| Expected in viral encephalitis | NA^c^ | ↑/N^d^ | ↑/N | ↑ | ↑ | ↑ | ↑ | ↑ | N | NA | ↑ |  |
| Expected in autoimmune encephalitis | NA | N | N | ↑ | NA | NA | NA | NA | NA | NA | N |  |
| a: all concentrations are given in pg/ml, b:concentrations marked by an asterisk are above the reference range, c: NA = not available, d: ↑ = elevated, N = normal | | | | | | | | | | | | |
