# Supplementary material for: Novel Orthobunyavirus Identified in the Cerebrospinal Fluid of a Ugandan Child With Severe Encephalopathy
Source: Clin Infect Dis. 2018 Jun 9;68(1):139–42. doi: 10.1093/cid/ciy486 (PMC6293039; doi:10.1093/cid/ciy486)
Supplement: Supplementary Table 4 [file ciy486_suppl_supplementary_table_4.doc]

| **Supplementary Table 4** – Oligonucleotide sequences of primers and probe used in this study | | |
| --- | --- | --- |
| **Name** | **Orientation** | **Oligonucleotide sequence (5´-3’)** |
| L4RU | Reverse | AARTGGAGTGCHCARGATGT |
| F1FU | Forward | AACAGAATTTGACATAACTCAG |
| F2FU | Forward | GAGATCTCTGAATTCCATCTGG |
| F3FU | Forward | GTTTCAATAAAAGAATACATC |
| M1F | Forward | CCTTATGAGCAATGTTTTAC |
| M1R | Reverse | TCTCCCATCTCTTTCTTCTC |
| M2F | Forward | TATGAGCAATGTTTTACAGG |
| M2R | Reverse | CCCATCTCTTTCTTCTCTGG |
| S1F | Forward | CAGGTGCAACCGTTCAGAC |
| S1R | Reverse | TTATAATCTCTTTCTTGGTC |
| S2F | Forward | GGTGCAACCGTTCAGACCGG |
| S2R | Reverse | ATCTCTTTCTTGGTCAATTC |
| NtwetweF | Forward | AATAATTTCAGAAGGTGCCCAAC |
| NtwetweP | Forward | ACAGTCAGCACATTTACATCGTCAA |
| NtwetweR | Reverse | AGCTGTTCGAAGTTGCCTAC |
