# Supplementary material for: Novel Orthobunyavirus Identified in the Cerebrospinal Fluid of a Ugandan Child With Severe Encephalopathy
Source: Clin Infect Dis. 2018 Jun 9;68(1):139–42. doi: 10.1093/cid/ciy486 (PMC6293039; doi:10.1093/cid/ciy486)
Supplement: Supplementary Table 5 [file ciy486_suppl_supplementary_table_5.doc]

| **Supplementary table 5 –** Raw Luminex plasma biomarker concentrations | | | | | | | | | | | |
| --- | --- | --- | --- | --- | --- | --- | --- | --- | --- | --- | --- |
| **Patient** | **GM-CSF** | **IL-10** | **IL-4** | **IL-6** | **IL-8** | **IP-10** | **MCP-1** | **MIG** | **MIP-1alpha** | **MPO** | **TNF-alpha** |
| 1 | 532.1 | 175.9 | 732.48 | 853.35 | 124.32 | 249.88 | 249.84 | 222.58 | 36.95 | 33073.15 | 301.51 |
| 2 | 815.93 | 225.75 | 838.1 | 1379.34 | 110.86 | 199.52 | 265.42 | 386.57 | 56.98 | 27871.59 | 599.8 |
| 3 | 631.8 | 161.56 | 1046.79 | 1447.15 | 187.43 | 185.16 | 327.72 | 297.99 | 57.86 | 123330 | 161.22 |
| 4 | 809.9 | 387.3 | 1228.32 | 1358.1 | 197.64 | 495.69 | 381.37 | 430.79 | 66.71 | 67548.02 | 440.59 |
| 5 | 331.96 | 148.74 | 527.53 | 331 | 53.82 | 114.51 | 213.34 | 72.28 | 12.04 | 15552.71 | 48.69 |
| 6 | 364.17 | 82.38 | 146.05 | 297.29 | 37.7 | 83.06 | 109.46 | 115.59 | 25.47 | 12548.02 | 185.66 |
| 7 | 236.58 | 134.51 | 170.58 | 261.12 | 27.17 | 232.44 | 140.59 | 98.39 | 21.71 | 11203.13 | 116.05 |
| 8 | 160.54 | 658.05 | 61.68 | 413.51 | 45.25 | 414.52 | 498.5 | 292.03 | 28.81 | 52332.52 | 30.59 |
| 9 | 319.32 | 123.53 | 318.42 | 374.03 | 116.53 | 390.75 | 327.62 | 372.37 | 36.24 | 61850.73 | 92.72 |
| 10 | 58.51 | 21.36 | 9.2 | OOR < | 10.87 | 24.93 | 34.78 | 13.11 | 24.98 | 12630.83 | 16.46 |
| 11 | OOR^a^ < | 4.98 | OOR < | OOR < | 1.56 | 21.23 | 9.5 | 107.63 | 9.53 | 56917.52 | 7.35 |
| 12 | 8.27 | 5.62 | OOR < | OOR < | 1.6 | 15.69 | 19.4 | 5.49 | 1.32 | 49648.69 | 9.48 |
| 13 | 206.99 | 20.89 | 65.68 | 167.63 | 26.17 | 56.23 | 40.31 | 95.59 | 19.74 | 41632.33 | 48.47 |
| 14 | 177.05 | 6.78 | 96.33 | 319.74 | 27.1 | 72.3 | 75.9 | 140.17 | 11.49 | 83577.67 | 104.19 |
| 15 | 206.65 | 32.41 | 118.98 | 172.31 | 16.73 | 195.85 | 84.41 | 65.77 | 21.37 | 15130.09 | 52.46 |
| 17 | 36.6 | 13.02 | OOR < | OOR < | 12.99 | 174.6 | 260.97 | 108.35 | 22.5 | 53967.41 | 4.72 |
| 18 | 89.95 | 105.98 | 14.36 | OOR < | 6.34 | 70.84 | 65.51 | 200.94 | 16.76 | 56500.37 | 16.92 |
| 19 | 45.89 | 90.59 | 16.32 | OOR < | 2.47 | 93.95 | 98.81 | 125.15 | 13.29 | 38879.41 | 16 |
| 20 | 81.86 | 187.29 | 28.13 | 2.36 | 6.81 | 317.64 | 352.38 | 486.01 | 31.27 | 138200.1 | 9.01 |
| 21 | 38.71 | 183.24 | 12.81 | OOR < | 5.67 | 187.77 | 263.15 | 708.87 | 17.82 | 92218.15 | 14.61 |

a: OOR = Out of range
