# Supplementary material for: Novel Orthobunyavirus Identified in the Cerebrospinal Fluid of a Ugandan Child With Severe Encephalopathy
Source: Clin Infect Dis. 2018 Jun 9;68(1):139–42. doi: 10.1093/cid/ciy486 (PMC6293039; doi:10.1093/cid/ciy486)
Supplement: Supplementary Table 6 [file ciy486_suppl_supplementary_table_6.doc]

**Supplementary Table 6** – Optimal number of hierarchical clusters determined by three different internal validation scores

| **Test** | **Score** | **Optimal number of clusters** |
| --- | --- | --- |
| Connectivity | 8.9829 | 2 |
| Dunn | 0.9757 | 5 |
| Silhouette | 0.3495 | 2 |
