# Supplementary material for: Novel Orthobunyavirus Identified in the Cerebrospinal Fluid of a Ugandan Child With Severe Encephalopathy
Source: Clin Infect Dis. 2018 Jun 9;68(1):139–42. doi: 10.1093/cid/ciy486 (PMC6293039; doi:10.1093/cid/ciy486)
Supplement: Supplementary Table 7 [file ciy486_suppl_supplementary_table_7.doc]

| **Supplementary table 7** – Rand index scores for varying distance metrics in hierarchical clustering | | | | | | |
| --- | --- | --- | --- | --- | --- | --- |
|  | Euclidean | Maximum | Manhattan | Canberra | Binary | Minkowski |
| Ward | 0.2769119 | 0.2769119 | 0.276912 | 0.276912 | 0.15719 | 0.276912 |
| Single | 0.2769119 | 0.2769119 | 0.276912 | 0.034281 | 0.15719 | 0.276912 |
| Complete | **0.5796943** | 0.2769119 | **0.579694** | 0.276912 | 0.15719 | **0.579694** |
| Average | 0.2769119 | 0.2769119 | 0.276912 | 0.268097 | 0.15719 | 0.276912 |
| Mcquitty | 0.2769119 | 0.2769119 | 0.276912 | 0.276912 | 0.15719 | 0.276912 |
| Median | 0.2769119 | 0.2769119 | 0.276912 | -0.05127 | 0.15719 | 0.276912 |
| Centroid | 0.2769119 | 0.2769119 | 0.276912 | **0.731996** | 0.15719 | 0.276912 |
