# Supplementary material for: Novel Orthobunyavirus Identified in the Cerebrospinal Fluid of a Ugandan Child With Severe Encephalopathy
Source: Clin Infect Dis. 2018 Jun 9;68(1):139–42. doi: 10.1093/cid/ciy486 (PMC6293039; doi:10.1093/cid/ciy486)
Supplement: Supplementary Methods [file ciy486_suppl_supplementary_methods.docx]

**Supplementary Methods**

**Ethics**

Informed consent was obtained from the patient’s parents. This patient took part in a larger observational study which was approved by Makerere University School of Medicine Research and Ethics Committee (Reference HS 1893).

**Viral metagenomics**

VIDISCA next-generation sequencing (NGS) was performed as previously described [1], with some modifications. CSF and plasma were pooled, centrifuged for 10 min at 10,000× g, and the supernatant was treated with Turbo DNase (Ambion). Subsequently, nucleic acids were extracted by the Boom extraction method [18]. A reverse transcription reaction with Superscript II (Thermo Fisher Scientific) was performed using non-ribosomal random hexamers [2]. A second strand DNA synthesis was performed with 5 U of Klenow fragment (3′ to 5′ exo minus, New England Biolabs, Ipswich, MA, USA). Double-stranded DNA was purified by phenol/chloroform/isoamyl alcohol extraction and ethanol precipitation and digested with MseI (New England Biolabs). Adaptors with a multiplex identifier sequence (MID) were ligated to the digested fragments. Before PCR amplification, the fragments were purified with AMPure XP beads (Agencourt AMPure XP PCR, Beckman Coulter, Brea, CA, USA). Next, a 28-cycle PCR with adaptor-annealing primers was performed. The program of the PCR-reaction was: 30 sec at 98 °C and cycles of 10 sec at 98 °C, 75 sec at 65 °C, followed by 5 min at 65 °C and 10 min 4 °C using Q5 High-Fidelity DNA Polymerase (New England Biolabs). After purification with AMPure XP beads, the DNA was quantified with the Qubit dsDNA HS Assay Kit (Thermo Fisher Scientific). The Bioanalyser (High Sensitivity DNA Analysis kit, Agilent Genomics) was used to determine the average nucleotide length of the library. Following size determination and DNA copy calculations, 50 pM DNA were clonally amplified on beads using the Ion Chef System (Thermo Fisher Scientific). Sequencing was performed on an Ion Proton™ System (Thermo Fisher Scientific).

Two Illumina viral RNA sequencing libraries were prepared, one for CSF and one for plasma. The pre-treatment, extraction, reverse transcription and second strand synthesis methods were similar to the VIDISCA-NGS library preparation. After second strand synthesis, Agencourt AMPure XP Beads were used for purification. The purified dsDNA samples were randomly sheared using NEBNext dsDNA Fragmentase (New England Biolabs). Samples subsequently underwent end repair with DNA Polymerase I, Large (Klenow) Fragment (New England Biolabs) and an overhang was created with Klenow Fragment (3’ --> 5’ exo-) (New England Biolabs) to which NEBNext Multiplex Oligos for Illumina adapters (diluted 1:1000) (New England Biolabs) were ligated with T4 DNA Ligase (5U/µl) (Invitrogen). Short dsDNA fragments (<150bp) and unbound adaptors were removed using AMPure XP beads. Ligated adaptors were treated with USER enzyme (New England Biolabs) followed by a Polymerase Chain Reaction (PCR) using Q5 High-Fidelity DNA Polymerase (NEB) and primers included in the Illumina adapter kit. Removal of post-PCR small fragments was performed with AMPure XP beads. The purified DNA was quantified with a Qubit dsDNA HS Assay Kit (Thermo Fisher Scientific) and a Bioanalyser (High Sensitivity DNA Analysis kit, Agilent Genomics) was used to determine the average nucleotide length of the library. Paired-end sequencing was performed on the Illumina MiSeq platform.

**Sequence analysis**

The sequence reads from both VIDISCA-NGS and Illumina viral RNA NGS were translated into protein sequences and a search for viral sequences was performed using the UBLAST [3] algorithm of USEARCH v10.0.240 and DIAMOND v0.9.21 [4]. The NCBI eukaryotic viral Identical Protein Groups were downloaded in March 2018, clustered at 95% amino acid identity with CD-HIT v4.7 [5], and used as reference sequences.

**Genome walking**Primers used for genome walking were designed on conserved regions from a random selection of orthobunyaviruses after ClustalW alignment using Bioedit software version 7.2.5 [6]. Primer L4RU combined with 3 sequential semi-nested internal VIDISCA fragment primers (F1FU, F2FU and F3FU in respective order) were used successfully for genome walking of the L-segment. Internal VIDISCA fragment primers M1F and M1R with nested primers M2F and M2R were used for M segment genome walking. Similarly, primers S1F and S1R with internal primers S2F and S2R were used for S segment genome walking. Primer sequences are available in Supplemental Table 4.

**Sanger sequencing**

To assure that mistakes introduced while making the VIDISCA or Illumina library are not in the sequences submitted to GenBank, all sequences were confirmed via direct Sanger Sequencing of amplicons (generated with Q5 enzyme, NEB). PCR products were sequenced using BigDye terminator chemistry (BigDye Terminator v1.1 Cycle Sequencing Kit, Applied Biosystems). Sequences were analyzed using Codoncode Aligner Software (Version 6.0.2).

**Amino acid phylogeny**

Nucleotide sequences were translated into amino acid sequences and a single open reading frame could be identified for all segments. The amino acid sequences were aligned to other orthobunyaviruses obtained from Genbank using all available multiple sequence alignments available of the M-Coffee package on the T-coffee server [7]. MEGA7 software version 7.0.21 was used to find the best protein Maximum Likelihood (ML) complete deletion model between all available methods [8]. The LG + G model consistently predicted the lowest Bayesian Information Criterion scores for all segments and was used to infer the phylogenies with 1000 bootstrap replicates.

**Development of a revere transcription quantitative polymerase chain reaction (RT-qPCR)**

The RNA was reverse transcribed as described by Oude Munnink et al. [9]. An RT-qPCR was developed with forward primer (NtwetweF), 6-carboxyfluorescein (FAM)-labeled probe (NtwetweP) and reverse primer (NtwetweR) (Supplemental Table 4). A dilution series of a plasmid clone of the L-segment, which completely encompassed the RT-qPCR amplicon, was used for calibration. The real-time PCR was performed on a Rotor-Gene Q (Qiagen) platform with 45 cycles using the standard manufacturer's protocol.

**Inflammatory biomarker measurements**

The following inflammatory biomarkers were measured, using a Luminex assay (Invitrogen) on the BioPlex System 100 (BioRad Laboratories, Hercules, CA, USA) with standard manufacturer’s protocol, in CSF and plasma of the Ntwetwe virus patient and plasma of 20 reference cases: GM-CSF, IL-4, 6, 8 and 10, IP-10, MCP-1, MIG, MIP-1α, MPO, and TNF-α. To minimize intra- and inter-assay variation, all plasma samples were analyzed in duplicate on the same plate. All statistical analyses were performed using R version 3.4.4. Two unsupervised machine learning methods, hierarchical clustering and principal component analysis, were used for comparing the plasma inflammatory biomarker profiles.

Samples with a value below or above detection limit were given the lowest or highest measured concentration for that biomarker respectively. Concentrations were divided by the median expression per biomarker and subsequently log2 transformed to remove the order of magnitude variations and centre the data. The resulting expression levels indicate absolute fold change from the median expression per biomarker. These transformed data were used as input data for all following analyses. Hierarchical clustering was performed using R studio version 1.1.383 with packages flashClust version 1.01-2 and visualized using pheatmap version 1.0.8 (both available on CRAN, <https://cran.r-project.org>). The number of clusters and all available distance metrics and clustering methods were compared for optimal external validation (‘variation of information’ score and Rand index) with package clValid version 0.6-6 and internal validation (Connectivity, Dunn and Silhouette scores) with package fpc version 2.1-10 (both available on CRAN). The Wilcoxon Rank Sum test was performed to test for differences between individual biomarkers between the two groups of clustered patients as they were non-normally distributed. A principal component analysis (PCA) was modelled using R package stats version 3.4.3 (available on CRAN) with a probability ellipse of one standard deviation plotted for the CNS-infection and non-CNS infection groups. To understand which biomarkers explained the most variation of the entire PCA model, the explained variance (ev) per biomarker (BioM) considering all PCs was calculated, where l = loading, eig = eigenvalue and i = the n^th^ of 11 principal components:

$${ev}_{BioM}=\frac{\sum_{i=1}^{11} {l_{i}}^{2}{eig}_{i}}{\sum_{i=1}^{11} {eig}_{i}}$$

Adapted from <http://www.sthda.com/english/articles/31-principal-component-methods-in-r-practical-guide/112-pca-principal-component-analysis-essentials/>

**Selection of reference cases for plasma biomarker profile analysis**

The reference cases were African children with encephalopathy and a known aetiology of a non-CNS infection (e.g. a brain tumour, an intracranial bleed, and cerebral malaria) or a proven CNS infection, defined as the presence of clinical symptoms matching the syndrome and a pathogenic organism isolated from CSF. Cerebral malaria was regarded as a non-CNS infection because it is an intravascular infection which leads to brain pathology, i.e. the CNS is not infected itself.

**References**

1. de Vries M, Deijs M, Canuti M, et al. A sensitive assay for virus discovery in respiratory clinical samples. PLoS One **2011**; 6.

2. Endoh D, Mizutani T, Kirisawa R, et al. Species-independent detection of RNA virus by representational difference analysis using non-ribosomal hexanucleotides for reverse transcription.

3. Edgar RC. Search and clustering orders of magnitude faster than BLAST. Bioinformatics **2010**; 26:2460–2461.

4. Buchfink B, Xie C, Huson DH. Fast and sensitive protein alignment using DIAMOND. Nat Methods **2015**; 12:59–60.

5. Li W, Godzik A. Cd-hit: a fast program for clustering and comparing large sets of protein or nucleotide sequences. Bioinformatics **2006**; 22:1658–1659.

6. Thompson JD, Higgins DG, Gibson TJ. CLUSTAL W: improving the sensitivity of progressive multiple sequence alignment through sequence weighting, position-specific gap penalties and weight matrix choice. Nucleic Acids Res **1994**; 22:4673–4680.

7. Notredame C, Higgins DG, Heringa J. T-coffee: a novel method for fast and accurate multiple sequence alignment 1 1Edited by J. Thornton. J Mol Biol **2000**; 302:205–217.

8. Kumar S, Stecher G, Tamura K. MEGA7: Molecular Evolutionary Genetics Analysis Version 7.0 for Bigger Datasets. Mol Biol Evol **2016**; 33:1870–1874.

9. Oude Munnink BB, Jazaeri Farsani SM, Deijs M, et al. Autologous Antibody Capture to Enrich Immunogenic Viruses for Viral Discovery. PLoS One **2013**; 8:e78454.
