# Supplementary material for: Novel Orthobunyavirus Identified in the Cerebrospinal Fluid of a Ugandan Child With Severe Encephalopathy
Source: Clin Infect Dis. 2018 Jun 9;68(1):139–42. doi: 10.1093/cid/ciy486 (PMC6293039; doi:10.1093/cid/ciy486)
Supplement: Supplementary Results [file ciy486_suppl_supplementary_results.doc]

**Supplementary Results**

Raw Luminex results are available in Supplemental Table 5

**Selecting optimal hierarchical clustering method**

Using two clusters gave the optimal silhouette and connectivity scores and was subsequently chosen (Supplementary Table 6). The Canberra centroid method provided the best external validation in terms of Rand index, yet was not chosen because the Complete distance metric provided a more overall robust external validation among varying clustering methods. The Euclidean, Manhattan and Minkowski clustering methods with a complete distance metric gave identical clustering (Supplementary Table 7).

**Immunological plasma profile results**

Using hierarchical clustering, all reference cases with and without a CNS-infections could effectively be separated. One patient was misclassified in each cluster which provided a false discovery rate of 0.12. The Ntwetwe virus patient clustered with the CNS-infection group close to a patient with a bilateral intracerebral pyogenic abscess and a patient with rabies virus and CMV in CSF. In the PCA analysis, six out of eight patients with and nine out of twelve patients without a CNS infections fell within their respective one standard deviation probability range with no false positives, the Ntwetwe virus patient fell within the CNS infection probability ellipse. Thus, both methods confirmed that a CNS infection was most likely in terms of host immunologic response.
