# Supplementary material for: Novel Orthobunyavirus Identified in the Cerebrospinal Fluid of a Ugandan Child With Severe Encephalopathy
Source: Clin Infect Dis. 2018 Jun 9;68(1):139–42. doi: 10.1093/cid/ciy486 (PMC6293039; doi:10.1093/cid/ciy486)
Supplement: Supplementary Figure 1 [file ciy486_suppl_supplementary_figure_1.doc]

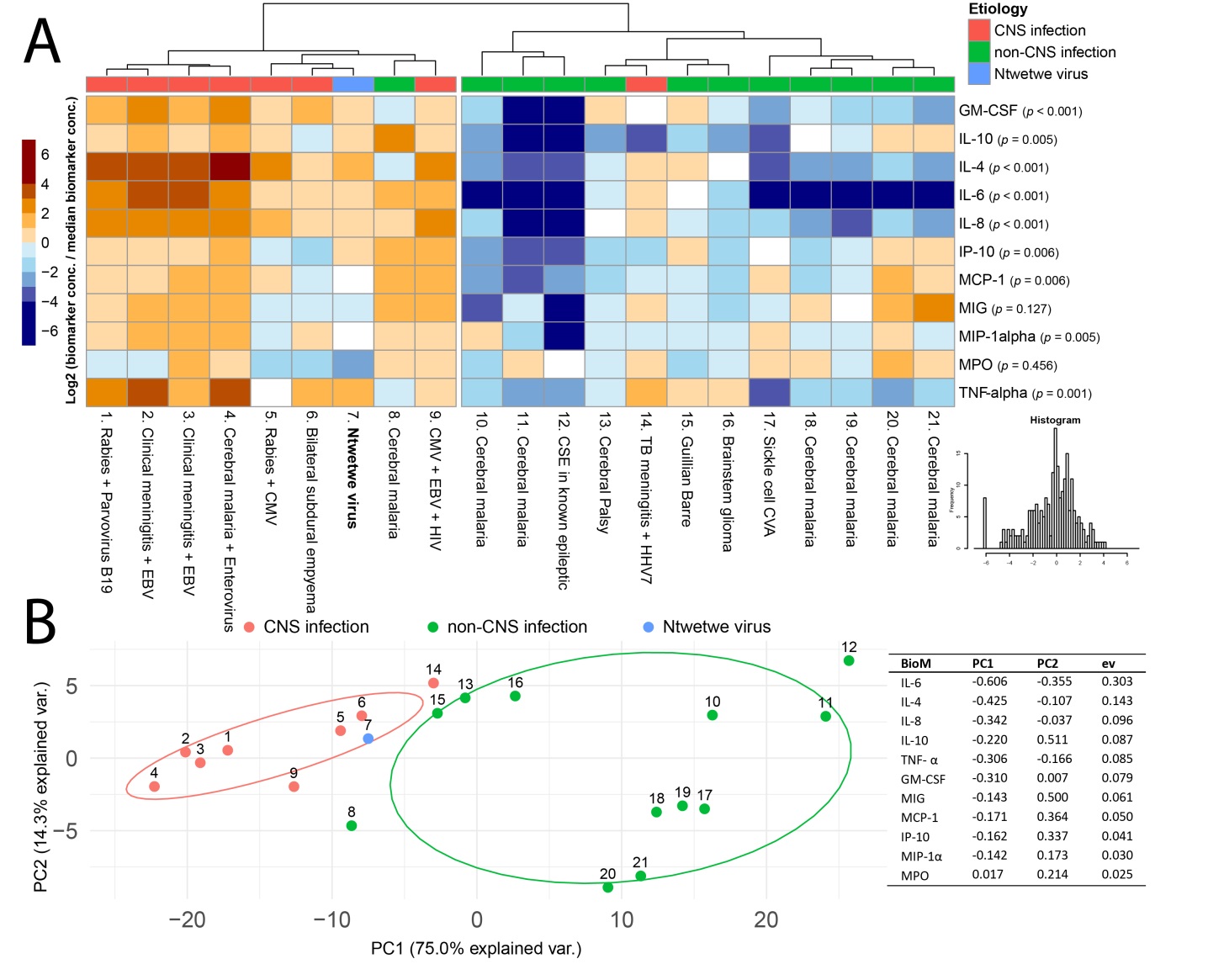


**Supplementary Figure 1.** Plasma immunological profiles of Ntwetwe virus patient and 20 reference patients with encephalopathy of varying aetiologies used for **A** hierarchical clustering. Values indicate concentration fold change from the median expression of all patients per biomarker where blue = below, white = equal to and orange = above median. Etiological group, diagnosis and results from a RT-qPCRs viral panel on CSF are shown for each patient. **B** principle component analysis. Principle component (PC) one and two are plotted which combined explain 88,7% of variance. Each sample (patient) is represented as a numerical unique identifier assigned in A. The table on the right depicts the loadings of the first two PCs for each tested biomarker (BioM) and the proportion of explained variance (ev) of each biomarker to the total variation of the model considering all PCs. EBV = Epstein-Barr virus, CMV = cytomegalovirus, HIV = human immunodeficiency virus, CSE = convulsive status epilepticus, HHV = human herpes virus, TB = tubercular, CVA = cerebrovascular accident
